# Does Intelligence Buffer or Amplify Personality Effects? An Experimental Test in a Brief Frustration-Induction Paradigm

**DOI:** 10.3390/jintelligence14100258

**Published:** 2026-10-13

**Authors:** Pei-Chun Shih, Daniel Peña, Daniel Wazne, Luis F. García, Mediss Tavakkoli

**Affiliations:** 1 Department of Biological and Health Psychology, Universidad Autónoma de Madrid, 28049 Madrid, Spain; dwazne@apertia-consulting.com (D.W.); luis.garcia@uam.es (L.F.G.); medisstavakkoli86@gmail.com (M.T.); 2 Grupo Alava Reyes, 28033 Madrid, Spain; dpena@apertia-consulting.com

**Keywords:** intelligence–personality interaction, process-level moderation, negative affect, mirror-tracing task

## Abstract

Progress on the intelligence–personality interface increasingly depends on studying the processes through which the two combine. One such process is whether intelligence buffers or amplifies personality expression under stress. Applied research reports such buffering for neuroticism on performance and conscientiousness on safety behavior, but not whether it extends to brief experimental contexts. We tested it with a mirror-tracing frustration paradigm with error-contingent penalties. Psychology undergraduates (*N* = 420 recruited; analytic *n* = 334–378) completed intelligence measures, personality inventories (NEO-FFI, BIS/BAS), and the PANAS pre- and post-task. The induction increased negative affect (*d* = 0.78). Neither hypothesis was supported, but the nulls are inconclusive: the design could detect only moderation well above the target magnitude, and equivalence tests could not exclude smaller effects. An exploratory analysis suggested, however, that intelligence may moderate a process rather than a trait: errors committed during frustration predicted affective change at higher levels of ability. This interaction held after residualizing errors on ability but did not survive Bonferroni correction and requires confirmatory replication. The buffering–amplifying question appears underspecified as posed; we propose an agenda specifying context, process, outcome, and required sample sizes.

## 1. Introduction

Understanding how intelligence and personality jointly shape individuals’ lives is a central aim of differential psychology, yet the two traditions have historically developed along parallel but largely separate paths (Ackerman & Heggestad, 1997; Cattell, 1987; Cuppello et al., 2023; DeYoung, 2020; Kulikowski & Ganzach, 2024). In a recent editorial outlining six key challenges for this field, Kulikowski and Ganzach (2024) argued that progress requires moving beyond demonstrating independent effects of intelligence and personality toward specifying the processes by which these constructs interact, including potential moderation effects. A fundamental question within this integrative agenda is whether intelligence buffers or amplifies the effects of personality on outcomes relevant to adaptation.

### 1.1. Evidence for Buffering Effects in Applied Settings

Two notable studies have reported evidence consistent with a buffering view of intelligence. Perkins and Corr (2006) examined the joint effects of cognitive ability and neuroticism on performance in military assessment centers. Across two samples (Naval *N* = 607; Army *N* = 62), neuroticism correlated negatively with performance only among individuals with lower cognitive ability; among those with higher cognitive ability, neuroticism was unrelated to performance. The authors proposed a cognitive buffering hypothesis: individuals with greater cognitive resources can absorb the cognitive load imposed by anxiety and still attend effectively to the task. Interaction effect sizes were modest (Δ*R*^2^ = 0.01 in Study 1; Δ*R*^2^ = 0.07 in Study 2), but consistent across samples.

Postlethwaite et al. (2009) extended this line of work to conscientiousness. Using a field sample of employees (*N* = 219) with supervisor ratings of workplace safety behavior, they found that conscientiousness predicted safety behavior only among individuals with lower cognitive ability; among those with higher cognitive ability, safety behavior was high regardless of conscientiousness level. Interaction terms accounted for 13–26% incremental validity in predicting safety behavior. Together, these studies suggest that intelligence may buffer the effects of both neuroticism (on performance) and conscientiousness (on rule-compliant behavior) in applied settings.

### 1.2. Evidence Suggesting Potential Amplifying Effects

A distinct literature suggests that intelligence may, under some conditions, amplify rather than buffer personality-linked vulnerabilities. Research on high-ability populations consistently identifies elevated performance expectations, perfectionism, and heightened sensitivity to failure as characteristic patterns (Fletcher & Speirs Neumeister, 2012; Guignard et al., 2012; Roedell, 1984; Stricker et al., 2020). Sweeny and Shepperd (2010) showed that students with more optimistic performance expectations reported greater negative affect after receiving feedback, controlling for actual performance.

The most developed mechanism is expectancy violation: higher-ability individuals may hold higher performance expectations, so that failure generates a larger discrepancy between expected and actual performance and, consequently, a stronger affective response (Mellers et al., 1997; Shepperd & McNulty, 2002). Closer self-monitoring of errors could contribute similarly. In either case, the emotional consequences of failure would depend on the magnitude of expectancy violation, and intelligence could amplify rather than buffer personality-linked reactivity to failure.

More recently, Robinson et al. (2023) found that higher cognitive ability, assessed via self-reported ACT scores, was associated with dampened emotional responding in a continuous-rating paradigm (the Dynamic Affect Reactivity Task): affective peaks were slower to arise and less pronounced, a difference in the dynamics of reactivity rather than in its global intensity. This pattern is consistent with dual-process accounts of affect regulation (Metcalfe & Mischel, 1999). Whether dampened reactivity to externally presented affective stimuli generalizes to contexts involving self-relevant performance failure is an open question.

### 1.3. The Role of Context

Whether intelligence buffers or amplifies personality effects may depend on the context in which personality expresses itself. Personality traits differ in the temporal and situational conditions under which they become behaviorally consequential. Conscientiousness, for example, is typically assessed through long-term goal pursuit (Roberts et al., 2014), and its predictive power may be weaker in brief laboratory tasks where state-level affective reactivity dominates. Similarly, neuroticism’s impact on performance may vary between sustained high-stakes situations (e.g., multi-day assessment centers; Perkins & Corr, 2006) and short laboratory tasks where momentary affective states are the primary outcome. A useful distinction may be drawn between slow trait expressions—requiring sustained effort, self-regulation, and engagement over time—and fast experimental contexts, in which dispositional tendencies have limited opportunity to manifest. As reviewed above, this evidence comes largely from applied contexts operating over longer time scales, raising the question of whether these effects generalize to brief experimental paradigms.

### 1.4. The Present Study

We examined intelligence–personality interactions in a controlled frustration-induction paradigm embedded within an individual-differences design. Frustration was experimentally induced, whereas intelligence and personality were measured and tested as moderators of affective and behavioral responses to the task. The paradigm, a mirror-tracing task, was well suited to this question for two reasons. First, it reliably increases negative affect in response to self-relevant performance difficulties, allowing the study of emotional reactions to failure under controlled conditions. Second, it yields continuous behavioral measures that permit the examination of behavioral responses under challenge. Because the task unfolds over a relatively short interval, it also provides a testbed for examining whether moderation effects previously documented in slower, applied contexts generalize to a brief laboratory setting. Given that the applied literature we sought to extend has primarily reported buffering effects, our confirmatory hypotheses addressed them. The amplification account, by contrast, is presented as an alternative theoretical possibility; it is examined not through a confirmatory test but through the exploratory analyses described below.

Two primary hypotheses were derived from Perkins and Corr (2006) and Postlethwaite et al. (2009), respectively. Because both derive from applied studies reporting small interaction effects (around ΔR^2^ = 0.01; Perkins & Corr, 2006), we expected moderation of comparably small magnitude.

**H1** (Affective domain)**.** *Based on Perkins and Corr (2006), we expected intelligence to buffer the effect of neuroticism on the change in negative affect from before to after the frustration task (ΔNA), such that the neuroticism–ΔNA association would be weaker among high-intelligence participants.*

**H2** (Behavioral domain)**.** *Based on Postlethwaite et al. (2009), we expected intelligence to buffer the effect of conscientiousness on behavioral persistence, such that conscientiousness would predict persistence more strongly among participants with lower intelligence. Persistence in this brief paradigm should be understood as a proximal behavioral analog of sustained goal-directed effort rather than a direct equivalent of the long-term conscientious behavior assessed in applied settings.*

These hypotheses were specified prior to data analysis. Beyond these primary tests, we conducted exploratory analyses to characterize the broader pattern of intelligence–personality interactions and to probe for process-level effects, in particular, whether intelligence moderates not the expression of traits but the translation of in-task performance into affective change. Exploratory findings are interpreted as hypothesis-generating rather than confirmatory and should not be viewed as a substitute for tests of the buffering hypotheses.

The contribution of this study is threefold. First, it provides one of the few experimental tests of intelligence as a moderator of personality effects on behavior and affect, complementing applied-setting findings. Second, it examines whether buffering effects generalize across contexts, addressing a boundary condition question. Third, in line with Kulikowski and Ganzach’s (2024) call for more process-oriented research on intelligence–personality interactions, it motivates reframing the buffering–amplifying question in context- and process-specific terms.

## 2. Materials and Methods

### 2.1. Participants

Four hundred and twenty undergraduate psychology students participated in exchange for course credit. The sample was one of convenience, its size determined by the number of students available rather than by an a priori power analysis. Of these, 404 completed the in-person intelligence session, 395 the online personality session, 392 the online PMA session, and 394 the online MTPT session; 378 provided complete pre- and post-task affect data. Cases were excluded analysis-by-analysis through listwise deletion whenever data were missing on any variable entering a given model; no participants were excluded for other reasons. Consequently, the analytic sample ranged from 334 to 378 depending on the variables involved in each analysis. The sample was predominantly female (*n* = 349, 85.5%), with a mean age of 19.04 years (*SD* = 4.22, range = 17–60). All participants provided informed consent. The study was approved by the institutional ethics committee.

Attrition occurred entirely between sessions. Of the 404 participants with available cognitive-ability scores, 394 initiated the mirror-tracing task, and all completed it. Participants who did not complete the mirror-tracing session did not differ from completers in cognitive ability (*d* = −0.05, *p* = .826), and no significant differences were observed at other stages of attrition (all |*d*| ≤ 0.31, all *p* ≥ .15). However, non-completers scored higher in neuroticism than completers (*M* = 40.13 vs. 36.17; *d* = 0.46, Welch’s *t* = 2.15, *p* = .038), whereas conscientiousness did not differ significantly between groups (*d* = −0.40, *p* = .121).

### 2.2. Materials

#### 2.2.1. Intelligence Measures

Intelligence was assessed using Raven’s Advanced Progressive Matrices (Raven et al., 1998) and three subtests of the Primary Mental Abilities battery (Verbal, Reasoning, and Spatial; Thurstone & Thurstone, 1941). A general factor of intelligence (FG) was computed as the standardized scores (*M* = 0.00, *SD* = 1.00) of the first unrotated principal component of these four measures, which accounted for 54.00% of the variance. Loadings ranged from 0.62 (Verbal) to 0.79 (Reasoning). FG was used as a continuous predictor in all analyses; interactions were probed at the mean and ±1 *SD* of FG and with the Johnson–Neyman technique (see Analytic Plan).

#### 2.2.2. Affect Measure

The Positive and Negative Affect Schedule (PANAS; Watson et al., 1988) was administered before and immediately after the mirror-tracing task using a validated Spanish adaptation (Sandín et al., 1999). The 20-item scale was divided into two fixed 10-item forms (each with 5 positive and 5 negative affect items): one administered before the task and the other after, to reduce item-repetition effects across the short interval. Items were rated on a 5-point scale with momentary (“right now”) instructions to capture state affect. Change in negative affect was computed as post-task minus pre-task NA (ΔNA).

#### 2.2.3. Personality Measures

The BIS/BAS Scales (Carver & White, 1994) measured behavioral inhibition and activation sensitivity. The NEO Five-Factor Inventory (NEO-FFI; Costa & McCrae, 1992) assessed Neuroticism, Extraversion, Openness, Agreeableness, and Conscientiousness. For both the NEO-FFI and the BIS/BAS Scales, scale scores were computed as the sum of their constituent items (after reverse-coding as appropriate). Both instruments were administered in validated Spanish adaptations (Caseras et al., 2003; Cordero Pando et al., 1999). Internal-consistency reliabilities for the personality measures are reported on the diagonal of Table 1. For the PANAS, Cronbach’s α was 0.76 (pre) and 0.77 (post) for negative affect and 0.80 (pre) and 0.83 (post) for positive affect.

#### 2.2.4. Mirror-Tracing Task

The task is a frustration-induction paradigm (Baumeister et al., 1998; Meindl et al., 2019; Quinn et al., 1996; Renna et al., 2018). We used an adapted version of the Mirror Tracing Persistence Task (MTPT), programmed ad hoc in the Phaser 2 web game-development framework (Photon Storm Ltd., Lydney, UK) and administered through the LimeSurvey platform (LimeSurvey GmbH, Hamburg, Germany). Participants traced figures with the computer mouse while the cursor movement was horizontally inverted (mirror effect), so that moving the mouse to the right displaced the cursor to the left and vice versa, creating a visuomotor conflict. The task comprised 12 trials organized into three phases of four trials each, using the same four figures of increasing difficulty throughout (a horizontal straight line, an “L” shape, an open circle, and a five-pointed star; see Figure 1). Within a session, the 12 trials were presented in a fixed sequence and could not be skipped: participants either advanced through them in order or discontinued the session entirely, with no option to bypass an individual trial and continue. The on-screen interface and its appearance across the three phases are illustrated in Figure 2. In the PRE phase (Trials 1–4), participants completed the figures without time limit or penalty, advancing automatically upon reaching 100% completion. In the EXP phase (Trials 5–8), designed to induce frustration, two modifications were introduced: each error returned the cursor to the starting point, and a visible countdown timer imposed time pressure (90 s for Trials 5–6 and 120 s for Trials 7–8); a trial ended either upon completion (highly unlikely, especially for the more complex figures) or when time expired. Instructions framed this phase as visuomotor-coordination training. The POST phase (Trials 9–12) reproduced the PRE conditions (no penalty, no time limit) and was framed as an assessment of improvement following the training, in order to mask the true purpose of the task and reduce participant reactivity. From trial-level data, we computed three behavioral indices averaged across the four EXP trials: Persistence, Restart Rate, and Task Completion. Persistence was computed as the proportion of trial time during which the participant was actively moving the cursor (active time divided by total trial duration, where total duration is the elapsed time of the trial). It ranges from 0 (no cursor movement) to 1 (continuous movement throughout), with intermediate values reflecting pauses and hesitations. Because participants almost never completed the EXP figures within the time limit, elapsed duration approximated the imposed limit in most trials. Restart Rate was computed as the number of errors divided by active time, yielding errors per millisecond, which we multiplied by 1000 for readability so that it is expressed as errors per second of active engagement. Task Completion was the percentage of the figure traced. Restart Rate indexes the frustration manipulation specific to the EXP phase: because each commission error (leaving the traced path) returned the cursor to the starting point, the number of errors equals the number of restarts. Behavioral data from the PRE and POST phases were recorded but are not analyzed here: the present study concerns responses during the frustration induction itself, and pre-post performance change indexes visuomotor learning, a distinct question that we do not address.

### 2.3. Procedure

Data were collected across four sessions distributed over approximately three weeks. In the first week, Raven’s Advanced Progressive Matrices was administered in person in a classroom setting, in groups of approximately 20 to 25 participants. The remaining sessions were completed online, and participants were given at least one week to complete each. In the second week, participants completed two online sessions: one comprising the personality questionnaires (NEO-FFI and BIS/BAS) and another comprising the three PMA subtests, administered with the standardized time limit for each subtest. In the third week, participants completed a final online session containing the full MTPT protocol with the pre- and post-task PANAS. This session also included additional cognitive tasks that are not analyzed in the present study. These additional tasks were administered before the pre-task PANAS and after the post-task PANAS, so that no task intervened between the two affect assessments other than the MTPT. Because access links were released sequentially by week, the Raven session preceded the personality and PMA sessions, which in turn preceded the MTPT session; within each release window, participants could complete the online sessions at a time of their choosing. Task parameters were identical for all participants, and the order of tasks and PANAS administrations was fixed rather than counterbalanced. Testing was conducted remotely and without direct supervision, and participants completed the protocol in their usual environments.

### 2.4. Analytic Procedures

The analytic strategy followed a primary-exploratory structure. Exploratory analyses examined: (a) whether FG moderated the relationship between in-task errors (Restart Rate) and ΔNA, and (b) the broader pattern of FG × personality interactions across all Big Five and BIS/BAS traits.

All analyses were conducted in IBM SPSS Statistics (Version 28; IBM Corp., Armonk, NY, USA). All predictor variables were mean-centered prior to computing interaction terms to reduce multicollinearity (Aiken & West, 1991). Statistical significance was set at *α* = 0.05 (two-tailed), with Bonferroni correction applied where appropriate for multiple comparisons.

Moderation hypotheses were tested using hierarchical multiple regression with mean-centered predictors and product terms. For H1, the model regressed ΔNA on FG, neuroticism, the FG × N interaction, and pre-task NA as a covariate. For H2, the model regressed persistence on FG, conscientiousness, and the FG × C interaction. The same approach was used for the exploratory FG × Restart Rate analysis on ΔNA, with progressively more demanding models adding neuroticism, BIS, and pre-task NA as covariates.

Significant interactions were probed using the PROCESS macro for SPSS (Model 1; Hayes, 2022; seed = 31,216), estimating conditional effects of the focal predictor at the mean and at ±1 SD of the moderator (Aiken & West, 1991), within the full model including all covariates. We additionally applied the Johnson–Neyman technique to identify the regions of the moderator continuum over which the conditional effect is statistically significant, avoiding the loss of information entailed by dichotomizing a continuous moderator.

For the focal exploratory interaction (FG × Restart Rate → ΔNA), we estimated a 95% percentile confidence interval for the interaction coefficient using a bootstrap with 5000 resamples, controlling for neuroticism, BIS, and pre-task NA. Conditional effects at the mean and ±1 *SD* of FG were estimated within PROCESS using its standard (normal-theory) confidence intervals.

For the systematic exploration of FG × personality interactions (28 tests across four outcomes and seven personality traits), we applied a Bonferroni correction for multiple comparisons, with adjusted *α* = 0.05/28 = 0.0018. Interactions not surviving this threshold are reported descriptively but not interpreted as confirmed. Because both the focal test and this sweep followed the null primary results, we report the focal interaction against both its uncorrected *p* value and the Bonferroni threshold rather than selecting a criterion post hoc.

Because the primary hypotheses concern moderation effects whose absence carries theoretical weight, we specified in advance how the evidential status of a null interaction would be evaluated. Three complementary criteria were used: sensitivity power analysis, the 95% confidence interval around each interaction coefficient, and an equivalence test. Together these criteria distinguish an informative null—an effect demonstrably smaller than the smallest magnitude of interest—from an inconclusive one.

Sensitivity analyses were conducted in G*Power 3.1 (Faul et al., 2009) using the F-test routine for linear multiple regression (fixed model, *R*^2^ increase), which returns, for each model, the smallest interaction effect (as Δ*R*^2^) detectable at a given sample size. For each primary model we specified *α* = 0.05, power = 0.80, one tested predictor, the analytic sample size, and the total number of predictors: *N* = 344 with four predictors for H1 and *N* = 359 with three predictors for H2.

Equivalence was assessed with two one-sided tests (TOST; Lakens et al., 2018). The smallest effect size of interest was set at Δ*R*^2^ = 0.01, the smaller value of interaction magnitude reported by Perkins and Corr (2006) (Study 1, Naval sample, *N* = 607). Although this effect was observed for performance rather than affective outcomes, we adopted it as a conservative reference point. Bounds were placed on the unstandardized interaction coefficient: for each model we converted the SESOI into an *F* value under the model’s residual variance, *F*(SESOI) = Δ*R*^2^/[(1 − *R*^2^)/*df*_residual_], and set the equivalence bound at *B* = *SE*(*B*) × √*F*(SESOI). The interaction coefficient was tested against each bound in turn, with the TOST *p* value taken as the larger of the two one-sided *p* values.

Analyses used listwise deletion within each model. Sample sizes ranged from *n* = 334 (full model with all covariates) to *n* = 378 (manipulation check), depending on the variables involved. Because attrition occurred almost entirely between sessions rather than during the analytic phase, and because any resulting range restriction would attenuate rather than inflate the hypothesized effects, listwise deletion within each model does not bias the tests toward significance.

## 3. Results

### 3.1. Preliminary Analyses

Descriptive statistics and intercorrelations for the intelligence and personality measures, together with internal-consistency reliabilities for the personality measures, are presented in Table 1.

### 3.2. Manipulation Check

Paired-samples *t*-tests confirmed that the induction produced the intended affective response. Negative affect increased from pre- (*M* = 1.81, *SD* = 0.80) to post-task (*M* = 2.43, *SD* = 0.96), *t*(377) = 14.79, *p* < .001, *d* = 0.78. Positive affect did not change significantly (*p* = .14).

### 3.3. Main Effects

Across the four EXP trials, Persistence averaged 0.37 (*SD* = 0.11, range 0.02–0.72), Restart Rate 0.35 errors per second of active time (*SD* = 0.49), and Task Completion 41% (*SD* = 20.19). Zero-order correlations use all available cases (pairwise deletion), as in Table 1. The regression models that follow use listwise deletion and therefore smaller samples. Higher intelligence (FG) predicted greater persistence (*r* = 0.21, *p* < .001), fewer errors (*r* = −0.18, *p* < .001), and higher task completion (*r* = 0.27, *p* < .001). Neuroticism predicted ΔNA (*r* = 0.12, *p* = .026) and lower persistence (*r* = −0.19, *p* < .001). Conscientiousness was unrelated to persistence in this sample (*r* = −0.036, *p* = .50).

### 3.4. Primary Analyses: Tests of the Buffering Hypotheses

Before presenting the primary tests, we note that the analytic samples provided limited statistical power for detecting the target effect. Sensitivity analyses indicated that the models could detect interaction effects of Δ*R*^2^ ≥ 0.022 with 80% power, roughly twice the magnitude reported in the source literature (Δ*R*^2^ = 0.01; Perkins & Corr, 2006). The primary results below should therefore be interpreted in the context of this methodological constraint.

H1: FG × Neuroticism → ΔNA

The model predicting ΔNA from FG, neuroticism, their interaction, and pre-task NA (*n* = 344) was significant overall (*R*^2^ = 0.150, *F*(4, 339) = 15.01, *p* < .001). The FG × Neuroticism interaction was not significant (*B* = −0.003, *SE* = 0.005, *t* = −0.65, *p* = .514, 95% CI [−0.012, 0.006], Δ*R*^2^ = 0.001). H1 was not supported.

This null is inconclusive rather than informative. Consistent with the power constraint noted above, the equivalence test did not exclude an effect of the target magnitude (*p* = .082; equivalence bound ±0.0100), and the confidence interval remains compatible with moderation in either direction. The data thus neither support nor refute intelligence-based buffering of neuroticism at the magnitudes documented in applied settings. What they do establish is the amount of evidence required to test this hypothesis adequately: experimental studies need substantially larger samples than have typically been used.

H2: FG × Conscientiousness → Persistence

The model predicting persistence from FG, conscientiousness, and their interaction (*n* = 359) showed a significant main effect of FG but no effect of conscientiousness (*B* = −0.001, *p* = .537), and no significant interaction (*B* = 0.001, *SE* = 0.001, *p* = .259, 95% CI [−0.001, 0.003], Δ*R*^2^ = 0.003). H2 was not supported.

Two features constrain this result. First, as with H1, the null is inconclusive: the model could detect interactions of Δ*R*^2^ ≥ 0.022 with 80% power, and the equivalence test was non-significant (*p* = .214; equivalence bound ±0.0016). Second, and more fundamentally, the expected conscientiousness–persistence association was, under these task conditions, not detectable for intelligence to moderate.

### 3.5. Exploratory Analyses

Process-Level Moderation: FG × Errors → ΔNA

Given the null primary findings, we tested a process-level hypothesis: that intelligence moderates not the expression of traits, but the translation of in-task failure into affective change. Specifically, we asked whether FG moderated the relationship between errors committed during the frustration phase (Restart Rate) and ΔNA. In the full model, controlling for neuroticism, BIS, and pre-task NA (*n* = 334), the FG × Restart Rate interaction was significant (*B* = 0.212, *SE* = 0.092, *t* = 2.30, *p* = .022, Δ*R*^2^ = 0.014, 95% CI [0.031, 0.393]). A percentile bootstrap with 5000 resamples returned the same coefficient with a slightly wider interval that likewise excluded zero (*B* = 0.212, bootstrap *SE* = 0.102, 95% CI [0.050, 0.447], *p* = .030).

Conditional effects from the model (Hayes, 2022) indicated that ability moderated the relationship between errors and change in negative affect. At +1 SD of FG, errors predicted increased negative affect (*B* = 0.361, *SE* = 0.152, *t* = 2.37, *p* = .018, 95% CI [0.062, 0.661]), whereas the effect was not significant at the mean (*B* = 0.148, *p* = .115) or at −1 SD of FG (*B* = −0.066, *p* = .541). A Johnson–Neyman analysis showed that the effect was significant for FG values above 0.274, representing 39.8% of the sample (Figure 3); that is, errors were associated with greater increases in negative affect only among participants at relatively higher levels of ability. This Johnson–Neyman value should be interpreted as the sample-specific point at which the effect becomes statistically significant, not as a substantive ability threshold.

Three robustness checks support this pattern. First, the interaction is stable across covariate sets: it holds in the basic model (*B* = 0.217, *p* = .027) and under progressively more demanding specifications adding neuroticism, BIS, and pre-task NA. Second, the finding does not depend on the normal-theory standard error, since the percentile bootstrap interval reported above also excludes zero. Third, the interaction is unlikely to be explained solely by the linear dependence between predictor and moderator. Restart Rate is itself correlated with ability (within the focal model’s sample, *r* = −0.169, *p* = .002): more able participants make fewer errors. To assess whether the interaction depended on this predictor–moderator overlap, we residualized Restart Rate on FG, leaving only errors committed above or below what would be expected given a participant’s ability, and by construction uncorrelated with it (*r* = 0.000). The interaction remained significant and, if anything, strengthened (*B* = 0.108, *SE* = 0.046, *t* = 2.38, *p* = .018, Δ*R*^2^ = 0.014), with the same conditional pattern (+1 *SD*: *B* = 0.182, *p* = .015; −1 *SD*: *p* = .483) and a Johnson–Neyman boundary at FG > 0.216.

Because the focal interaction followed the null primary results, we report it against both its uncorrected *p* value and the sweep’s Bonferroni threshold (α = 0.0018) rather than selecting a criterion after the fact and treat it as suggestive rather than confirmatory.

Exploratory FG × Personality Interactions on Behavioral Outcomes

We tested all FG × personality interactions (Big Five + BIS/BAS) on behavioral outcomes (Persistence, Restart Rate, Task Completion) and ΔNA. Of 28 tests, five reached *p* < .05, all involving Extraversion (E) and Agreeableness (A) on behavioral outcomes. After applying a Bonferroni correction for multiple comparisons (adjusted *α* = 0.05/28 = 0.0018), none of these interactions formally survived correction. No FG × personality interaction on ΔNA approached significance (all *p* > .37).

These nominal interactions are summarized in Appendix A Table A1. In brief, the five nominally significant effects all involved Extraversion or Agreeableness predicting behavior at lower levels of FG but not at higher levels. These effects do not survive correction; they were not predicted, and, unlike the process-level interaction above, they have not been subjected to robustness checks. We report them for transparency and completeness and note only that whether personality traits lose behavioral relevance at higher ability is a question that would need to be posed prospectively, with adequate power, rather than recovered from an exploratory sweep.

## 4. Discussion

We asked whether the buffering effects documented in applied settings (Perkins & Corr, 2006; Postlethwaite et al., 2009) extend to a brief experimental frustration paradigm. Neither primary hypothesis was supported. The contribution is not the nulls themselves but what they let us specify. Sensitivity analyses show this design could detect only moderation effects more than twice the size of those it sought to extend, so the primary nulls are inconclusive by construction, and we say so. What the study does deliver is that intelligence moderated something in this paradigm, though not what the trait-level hypotheses predicted: it moderated the translation of in-task failure into affective change. That finding, together with the evidential boundaries the nulls establish, points toward a more specific formulation of the buffering–amplifying question.

### 4.1. What the Null Findings Establish

The primary nulls cannot be used to claim that intelligence fails to buffer neuroticism or conscientiousness in brief experimental contexts: the equivalence tests did not reject effects of the magnitude reported in the source literature, and the confidence intervals remain compatible with moderation in either direction. The same caution applies within the affective model: neuroticism and intelligence each predicted affective change, but we detected no interaction between them, and a non-significant interaction is not evidence that their effects are additive, only that no moderation was detectable at this sample size. What they do establish is a quantitative constraint the applied literature has not made explicit. Detecting moderation of the size Perkins and Corr (2006) report, Δ*R*^2^ = 0.01, in an experiment requires roughly 750–800 participants for 80% power, more than double what we recruited, and far more than is typical in laboratory work. Investigators who wish to test intelligence-based buffering now have a concrete number to plan against. Such constraints become visible only when nulls are reported together with their sensitivity analyses.

One null here is genuinely informative, though not the one we hypothesized: conscientiousness showed no detectable association with persistence. This is not a power problem, since the effect is bounded well below anything of substantive interest. Nor is it an artifact of the measure: persistence varied widely across participants, so the null does not reflect a floor or ceiling on the dependent variable. Conscientiousness is a disposition toward sustained, goal-directed effort (Roberts et al., 2014), and Postlethwaite et al. (2009) found substantial effects on supervisor-rated safety behavior, a criterion accumulated over months. An eight-minute mirror-tracing task offers no comparable opportunity for that disposition to express itself; here, behavior was driven by state-level affective reactivity instead, with neuroticism predicting persistence and conscientiousness not. H2 was, in retrospect, a poor test of the buffering account. We do not conclude that the association is absent; we conclude that this paradigm, over this interval, did not elicit it, and that a buffering test built on it therefore had nothing to work on.

We therefore propose stressor duration as a candidate dimension: the interval over which a dispositional tendency can accumulate behavioral consequences. The prediction is ordinal and falsifiable, in that buffering effects should increase with duration, and a design that manipulates duration while holding population, task, and outcome constant would test it. We formulated this conjecture after seeing our results, not before. Our contexts also differ from the applied studies in ways confounded with duration, among them population, evaluative stakes, ability range, and outcome type, any of which could carry the difference. Duration is the dimension we consider most tractable to manipulate, not the one we have shown to be responsible.

### 4.2. A Process-Level Moderation

Where the trait-level hypotheses found nothing, a process-level test found something. Intelligence did not change how much neuroticism mattered; it changed how much errors mattered. Among participants above roughly a quarter of a standard deviation in ability, some 40% of the sample, committing more errors during the frustration phase predicted a larger increase in negative affect. Below that threshold, errors and affect were unrelated. The shape of the effect constrains its interpretation. What ability changed was the slope relating errors to affect, not the level of distress reached once errors were many. Predicted affective change among more able participants was lowest when errors were few and rose steeply as errors accumulated, converging on the values observed at lower ability rather than exceeding them. In this paradigm, then, ability was associated with an affective advantage that failure eroded, rather than with a heightened vulnerability to failure as such.

The most serious objection to such a finding is that it might be structural rather than psychological. Errors are not independent of ability, since abler participants make fewer of them, so an interaction between ability and error count could simply reflect that a given number of errors means something different at different ability levels. We tested this directly. Residualizing errors on ability leaves only the discrepancy between performance and what ability would predict, a quantity uncorrelated with ability by construction, and the interaction survives that transformation. The moderation is therefore not accounted for by the linear dependence between ability and error rate. Notably, the residualized discrepancy has no main effect on affect (*p* = .393): performing below what ability predicts was associated with affective change only among higher-ability participants. The effect is small and exploratory, and does not survive the sweep’s Bonferroni threshold; we do not treat it as confirmatory. It remains useful as a source of testable hypotheses, being directionally coherent, stable under each check we applied, and specific enough for a confirmatory study to falsify.

Several mechanisms would produce this pattern, and the present design cannot distinguish them. Expectancy violation is the most developed candidate (Burgoon, 2015; Mellers et al., 1997; Sweeny & Shepperd, 2010): higher ability may confer higher performance expectations (Chemers et al., 2001; Paunonen & Hong, 2010), so that failure generates a larger discrepancy and a sharper affective response, a mechanism for which Sweeny and Shepperd (2010) provide direct evidence, showing that students with more optimistic exam expectations report greater negative affect after feedback, controlling for actual performance. But differential self-monitoring (abler participants tracking their errors more closely) and differential task investment (abler participants engaging more, and so feeling failure more acutely) predict the same interaction. We measured none of these, so we advance expectancy violation as the interpretation we find most plausible, not as the one the data select. The asymmetry noted above bears on this. An account in which the affective consequence of failure depends on what was expected of oneself accommodates this asymmetry more naturally than one in which failure is simply aversive. The finding also bears on Robinson et al. (2023). Reactivity to externally presented stimuli and reactivity to self-relevant failure may be different processes that move in opposite directions with ability, a possibility open to direct test.

### 4.3. Toward a Context- and Process-Specific Agenda

The question this study set out to answer, whether intelligence buffers or amplifies personality effects, presupposes a single answer. Within one paradigm and one sample we found no trait-level moderation and an exploratory, process-level pattern in which the affective advantage associated with higher ability appeared to diminish as errors accumulated. The answer, it appears, may depend on what is being moderated.

We therefore propose decomposing the question rather than answering it, along three parameters. The first is level: whether intelligence moderates the expression of a trait or the processing of an experience. These are different claims, and disentangling them is one thing our design was able to begin. The second is timescale: advanced above as the duration conjecture and specified so as to be falsifiable. The third is outcome modality: Perkins and Corr found buffering on assessor-rated performance; we found none on self-reported affect, but observed an exploratory process-level pattern in the error–affect link. These may be answers to different questions rather than inconsistent answers to one. We offer this as a tentative agenda rather than a framework. It rests on a single underpowered study with an exploratory finding, and we present it to guide future tests rather than as a conclusion these data establish.

### 4.4. Contributions

The study contributes on three fronts. It brings an experimental design to a question studied almost entirely in applied, correlational settings: the stressor is manipulated rather than observed, and the behavioral indices are generated by the task itself. That is what makes a process-level moderation detectable at all, and it answers Kulikowski and Ganzach’s (2024) call for process-focused research. It quantifies the evidential requirements of the question rather than merely failing to answer it: by pairing null interaction tests with sensitivity analyses, confidence intervals, and equivalence tests, we distinguish an effect that is absent from one that is undetectable at this sample size, and we report the sample size at which it would become detectable. And it identifies a candidate process: a moderation not of a trait but of the link between failure and affect, located on the ability continuum rather than dichotomized, and holding when errors are residualized on ability, which addresses the structural artifact of predictor–moderator dependence. We offer it as a hypothesis, but one with a specific shape, a specified boundary, and a clear falsification condition.

These findings, though preliminary, are consistent with integrative models of intelligence and personality aimed at understanding behavioral outcomes (Colom et al., 2019; Stanek & Ones, 2023). If replicated, this pattern suggests that assessing cognitive ability may help explain why individuals with similar personality profiles respond differently to the same stressor, not because ability blunts a trait, but because it may condition how failure is translated into affect. At present, however, we view this as a hypothesis for future investigation rather than a conclusion that can be drawn from the current evidence.

### 4.5. Limitations

The principal limitation is statistical power, which we have quantified rather than merely acknowledged: the primary models could detect moderation only well above the target effect, so the hypotheses were tested against a standard they could not meet. Two features of the sample compound this. Psychology undergraduates are positively selected on ability and relatively homogeneous in personality, and restricted variance attenuates observable interactions (Hunter & Schmidt, 2004; Sackett & Yang, 2000); the sample was also predominantly female, so, beyond its effect on power, this composition limits generalization to more balanced or non-student populations, and replication in more heterogeneous community samples is warranted. Selective attrition worked in the same direction: those who did not attend the task session scored higher in neuroticism, truncating the upper tail of the variable H1 concerns. Cognitive ability showed no attrition bias.

Within the task there was no attrition at all. Every participant who began the mirror-tracing task completed all twelve trials, and because trials could not be skipped, completing them required staying through the aversive phase while disengaging remained available throughout. This concerns participation rather than effort, since participants varied widely in how actively they engaged, but it establishes that the induction did not drive participants out of the study. The primary nulls cannot be attributed to differential dropout during the frustrating phase.

Three further constraints bound what can be claimed. The focal interaction is small, exploratory, and unreplicated. The expectancy-violation account is one of several consistent with the data, and we measured none of the candidate mediators. And we studied one induction paradigm over one brief interval; whether these patterns hold under sustained challenge is what the duration conjecture proposes to test, and this design cannot settle it.

The measures bound the interpretation further. Residualizing Restart Rate on FG removes the component of error rate attributable to ability, but the residual is not thereby a pure index of expectancy discrepancy: errors above what ability predicts may also reflect impulsivity, inattention, motor control, or risk tolerance, none of which we measured. The residualization addresses the concern that the interaction is structurally entailed by predictor–moderator dependence; it does not establish what the residual represents. Because negative affect was assessed only once, after all twelve trials, our conclusions concern affective responses within a frustration-induction context rather than frustration as a discrete state. A further measurement constraint is that the pre- and post-task PANAS comprised two fixed item halves rather than counterbalanced sets, so the change score confounds affective change with differences between the item sets. Because this difference is constant across participants, it may bias estimates of mean-level change but cannot account for the between-person variation in change scores or the associations on which the focal interaction depends.

### 4.6. Future Directions

Three lines follow directly. A properly powered test of the trait-level hypotheses is the first: our sensitivity analyses indicate roughly 750–800 participants would be needed to detect, experimentally, moderation of the magnitude the applied literature reports. Until that study is run, whether intelligence buffers neuroticism in brief contexts remains open rather than answered in the negative. The second is a test of the duration conjecture. Because the prediction is ordinal, a single design can manipulate duration while holding population, task, and outcome constant, unconfounding duration from the population and stakes differences that separate our study from the applied ones. The third is confirmatory replication of the process-level moderation with the candidate mechanisms measured rather than inferred; samples spanning the full ability range would also establish whether the Johnson–Neyman boundary we identify is a property of the process or of our sample.

## 5. Conclusions

Intelligence and personality did not interact in the ways the applied literature led us to expect, but we are not in a position to say they never do. This design could not have detected effects of the size that literature reports, and we have quantified how much larger a study would need to be. That constraint is the first thing a reader should take from this paper. The second is that the exploratory analyses suggest a possible role for intelligence in moderating the link between in-task errors and affective change at relatively higher levels of ability, a pattern that now requires confirmatory replication.

Whether intelligence buffers or amplifies personality effects may be, as posed, a question without an answer, since it specifies neither context, nor outcome, nor process, nor timescale. The more productive question is when, for what, and through what mechanism ability shapes the way dispositional tendencies translate into affective and behavioral outcomes.

## Figures and Tables

**Figure 1:**
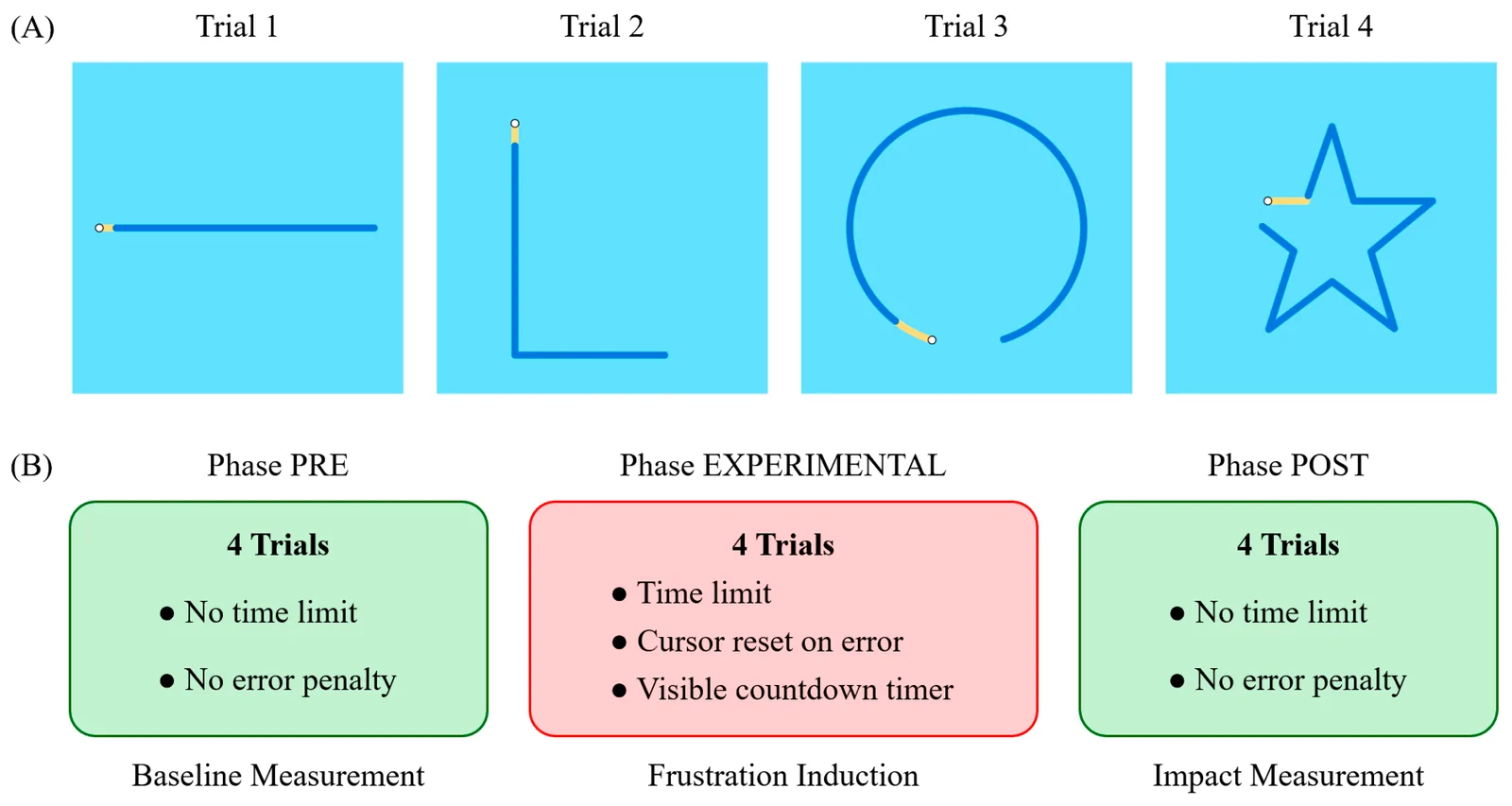
Stimuli and phase structure of the mirror tracing persistence task. Note.(**A**) The four figures traced in the task—a horizontal line, an “L”, an open circle, and a five-pointed star—in order of increasing difficulty. (**B**) The three phases of the task: the PRE phase (baseline measurement), the EXPERIMENTAL phase (frustration induction), and the POST phase (impact measurement), each comprising four trials under the conditions shown.

**Figure 2:**
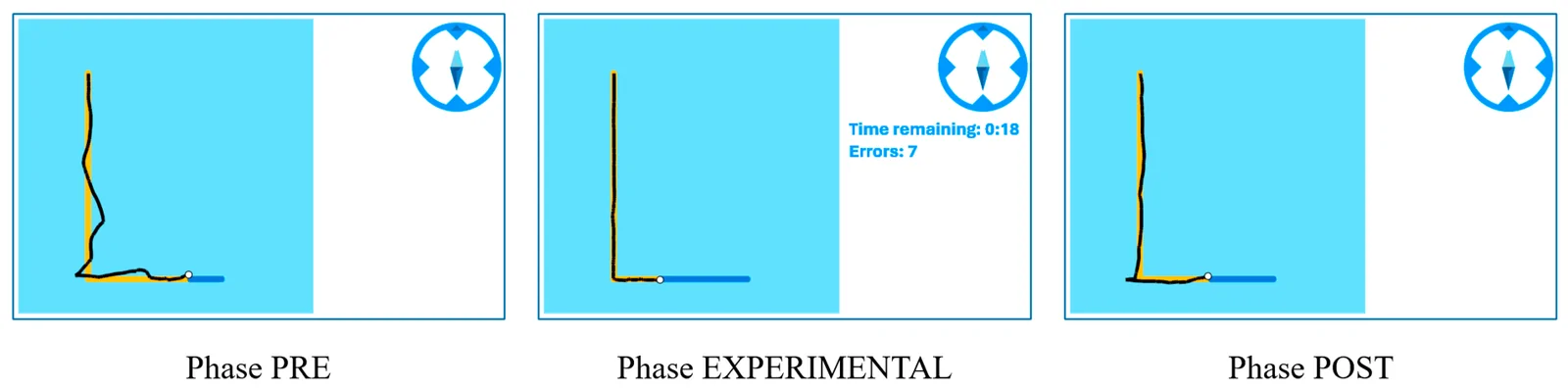
On-screen interface of the mirror tracing persistence task across the three phases. Note. The task interface in the PRE, EXP, and POST phases. The countdown timer and error counter are visible only in the EXP phase.

**Figure 3:**
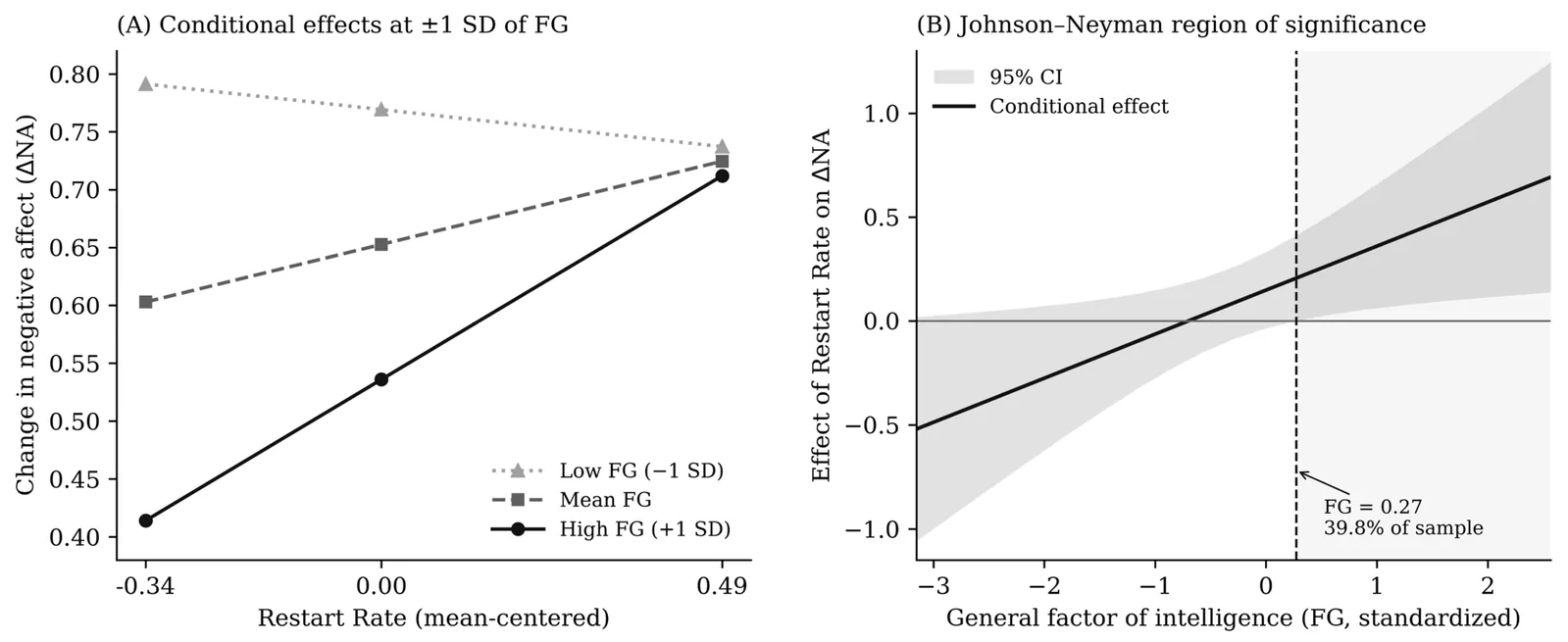
The moderating role of intelligence in the relationship between in-task errors and affective change. Note. (**A**) Conditional effects of Restart Rate on change in negative affect (ΔNA) at −1 *SD*, the mean, and +1 *SD* of the general factor of intelligence (FG). (**B**) Johnson–Neyman plot of the conditional effect of Restart Rate on ΔNA across the FG continuum, with 95% confidence bands. The effect becomes statistically significant at FG > 0.274 (dashed line), corresponding to 39.8% of the sample; the shaded region marks the region of significance. Both panels derive from the full model (*n* = 334), controlling for neuroticism, BIS, and pre-task negative affect; Restart Rate is mean-centered.

**Table 1:** Descriptive statistics, reliabilities, and intercorrelations for intelligence and personality measures.

Variable	*M*	*SD*	1	2	3	4	5	6	7	8
1. FG	0.00	1.00	-							
2. Neuroticism	36.33	8.56	−0.05	0.85						
3. Extraversion	43.49	7.73	0.00	−0.28 ***	0.85					
4. Openness	44.34	6.83	0.04	0.02	0.15 **	0.75				
5. Agreeableness	41.44	6.01	−0.08	−0.13 **	0.22 ***	0.13 **	0.68			
6. Conscientiousness	44.25	7.24	−0.05	−0.26 ***	0.07	0.03	0.08	0.84		
7. BIS	22.17	3.37	0.00	0.54 ***	−0.20 ***	−0.03	0.18 **	−0.05	0.79	
8. BAS	39.53	4.56	−0.11 *	0.02	0.39 ***	0.23 ***	0.01	0.10 *	−0.01	0.75

Note. *N* = 366–395 (pairwise deletion; all available cases). FG = general factor of intelligence (first unrotated principal component; standardized). Personality scores are sum scores. Cronbach’s *α* reliabilities appear on the diagonal; FG is a component score and has no *α*. BIS = behavioral inhibition; BAS = behavioral activation. * *p* < .05. ** *p* < .01. *** *p* < .001.

## Data Availability

The datasets are not readily available because they form part of an ongoing broader research project; requests should be directed to the corresponding author.

## Abbreviations

The following abbreviations are used in this manuscript:

FG	General Factor of Intelligence
MTPT	Mirror Tracing Persistence Task
PANAS	Positive and Negative Affect Schedule
ΔNA	Change in Negative Affect
NEO-FFI	NEO Five-Factor Inventory
BIS/BAS	Behavioral Inhibition/Activation System
PMA	Primary Mental Abilities
TOST	Two One-Sided Tests
SESOI	Smallest Effect Size of Interest

## Appendix A

**Table A1:** Exploratory FG × Personality Interactions Reaching Uncorrected *p* < .05.

Interaction	Outcome	*B*	*p*
FG × Extraversion	Persistence	−0.0021	0.004
FG × Extraversion	Restart Rate	0.0067	0.027
FG × Extraversion	Task Completion	−0.2640	0.038
FG × Agreeableness	Persistence	0.0030	0.004
FG × Agreeableness	Restart Rate	−0.0131	0.003

Note. Only interactions with uncorrected *p* < .05 are shown. None survives Bonferroni correction. No FG × personality interaction on ΔNA reached significance (all *p* > .37).
